# Outcomes of treatment with short dental implants compared with standard-length implants: a retrospective clinical study

**DOI:** 10.1186/s40902-024-00419-8

**Published:** 2024-02-28

**Authors:** Kinga Bérczy, György Göndöcs, György Komlós, Tatiana Shkolnik, György Szabó, Zsolt Németh

**Affiliations:** Faculty of Dentistry, Department of Oro-Maxillofacial Surgery and Stomatology, Semmelweis University, Mária Street 52, 1085 Budapest, Hungary

**Keywords:** Short implants, Standard implants, Success rate

## Abstract

**Background:**

The size of dental implants is a key success factor for appropriate osseointegration. Using shorter implants allows the possibility of avoiding complex surgical procedures and reduces the morbidity of treatment. Shorter implants also enable implant-prosthetic rehabilitation after maxillofacial reconstructions where only limited bone is available. In this study, the success rates of short implants were examined and compared to those of standard-sized implants.

**Methods:**

Patients who received dental implants between 2007 and 2016 at the Department of Oro-Maxillofacial Surgery and Stomatology Semmelweis University were enrolled in the study. Several clinical parameters were recorded and supplemented with radiological examinations. The data were statistically analysed.

**Results:**

Thirty-four patients with a total of 60 implants were included. The average time after prosthetic loading was 39.33 ± 21.96 months in the group with 8-mm implants and 41.6 ± 27.5 months in the group with > 8-mm implants. No significant differences were observed between the two groups in terms of probing depth (short implants, 2.84 ± 0.09 mm; standard implants, 2.91 ± 0.35 mm) or mean marginal bone loss (short implants, 1.2 ± 1.21-mm mesially and 1.36 ± 1.47-mm distally; standard implants: 0.63 ± 0.80-mm mesially and 0.78 ± 0.70-mm distally).

**Conclusions:**

In this study, the success rate of short dental implants was comparable to that of standard-sized implants. Consequently, it can be claimed that the long-term success of short dental implants does not differ significantly from the long-term success of standard implants.

## Background

In the 1980s, Albrektsson and Brånemark [1] described the necessary conditions to achieve optimal osseointegration. These factors included the following: implant material, adequate surgical technique, prosthetic loading, implant surface treatment, and implant size. The use of short implants raises questions in several areas. The primary stability, the bone-implant contact, the increased crown-implant ratio, and their combined effect on marginal bone loss, as well as the long-term success of the implant, are questionable. However, many factors have changed since the 1980s. Surface-treated implants appeared, new implant materials and different implant forms were developed, and new surgical techniques have been developed. However, the implant length and the definition of short implants are changing continuously. In the past, implants shorter than 10 mm placed in the lower jaw and shorter than 13 mm placed in the upper jaw were considered to be risk factors for implant success [2]. Furthermore, it was expected that short implants would have a lower success rate and an unpredictable survival rate. Even in the early 2000s, numerous publications described short dental implants as being less successful than standard implants of conventional length [3, 4]. Later, in 2016, at the European Association of Dental Implantologists (EDI) consensus meeting, a short implant was defined as being 8-mm long [5]. At the same meeting, the recommendation was issued that short implants should be a minimum of 3.75 mm in diameter. The term “ultrashort implant” has emerged, describing implants with a length of 6 mm. For ultrashort implants, there is insufficient evidence to make recommendations at this time. These definitions were confirmed in 2023 at the 18th EDI conference [6]. Publications from the end of the 2010s reported more favourable data on the success of short implants [7–9]. According to the current classification, the 8-mm-long implants included in our study were considered short, and implants larger than 8 mm were considered standard. The use of short implants has many advantages. Bone vertical augmentation, as well as sinus elevation procedures in the posterior maxilla directed to increase vertical dimensions, can be avoided. Furthermore, compared with implant placement combined with bone augmentation, there is a lower risk of damaging important anatomical structures, a lower morbidity, a shorter treatment time, and an overall lower cost [10–12]. The use of short implants can have advantages not only in the case of jaw atrophy but also in jaw reconstruction surgery involving the fibula or scapula because, due to limited bone volume, the use of a short implant better facilitates prosthetic rehabilitation [13, 14]. The aim of the present study was to clinically and radiographically assess the success of short and standard implants in a retrospective comparative clinical trial.

## Methods

### Study design

Patients who had received at least one dental implant during 2007–2016 at the Department of Oro-Maxillofacial Surgery and Stomatology Semmelweis University, Budapest, Hungary, were enrolled in the study. The inclusion criteria were as follows: no medical conditions that would adversely influence the long-term outcomes of implant therapy (such as diabetes mellitus and immunosuppressive status); nonsmoking; good oral hygiene; and at least 6 months of loading. The exclusion criteria were as follows: the use of systemic steroids, bisphosphonate therapy, pathological conditions at the study sites, misfitting of prosthetic components, malocclusions, bruxism, or no bone augmentation (neither at the same time as the implantation nor prior to the implantation).

The study protocol was approved by the National Institute of Pharmacy and Nutrition (reference number: Országos Gyógyszerészeti és Élelmezés-egészségügyi Intézet/29164/2019). The data collection was performed with the understanding and written informed consent of every participant. The study was conducted in full accordance with the Helsinki Declaration of 1975 [15], as revised in 2013 [16]. The patients were divided into two groups: (i) patients with 8-mm-long implants (test group) and (ii) patients with > 8-mm-long implants (control group). Implants with a length of 8 mm were considered short implants according to the latest guidelines of the European Association of Dental Implantologists [6]. Envelope or triangular mucoperiosteal flaps were prepared, and a two-phase surgical technique was used. In all cases, the implants had adequate primary stability (25–35 Ncm). The implants were exposed and loaded 3 months after the insertion.

### Data collection

For the radiographic analysis, *long-cone* intraoral radiographs were taken to identify mesial and distal marginal bone loss (MBL) as the primary outcome measure. MBL was measured either from the implant platform in the case of bone level (BL) implants or from the border of treated and polished implant surfaces in the case of tissue level (TL) implants. The following clinical parameters were assessed as secondary outcome measures: probing depth at six sites per implant (mesiobuccal, buccal, distobuccal, disto-oral, oral, mesio-oral), the *Silness–Löe plaque index* (SLPI), and bleeding on probing (BOP). Furthermore, the extent of tooth loss (total, partial, single); the type of prosthesis (single crown, fixed partial denture, full arch bridge); and the duration of prosthetic implant loading were also recorded. The following implant parameters were recorded: implant size, position, diameter, type, material, and surface.

### Statistical analysis

We followed a two-step approach of (i) exploratory data analysis [17] and (ii) a subsequent confirmatory data analysis. In the exploratory step, the relevant phenomena in the underlying data were verified at a high level. Pairwise correlations amongst the numeric variables were calculated, and the results were visually investigated. Additional graphical techniques were applied to gain insight into the characteristics of the data, such as their normality. In the confirmatory step, a test of equal values was carried out between the test and control group data. Having gained no relevant insight into the correlations between the variables in the prior step, the following independent variables were manually selected for the test:

- The type of implant (BL or TL);
- Observable BOP;
- The value of the SLPI;
- The type of tooth loss (free-end situation, complete edentulism in one arch, total edentulism in both arches).

Using these independent variables, Welch’s t tests of unequal variances were carried out. Welch’s t test is an adaptation of Student’s t test, with improved reliability for data samples with unequal variances and unequal sample sizes. Even though Welch’s test assumes a normal distribution of the underlying data, it is considered to be robust against deviations from normality.

The null hypothesis stated that the means of the two samples would not be significantly different. Upon observation of a *p* value that was above the chosen significance threshold of 0.05, the null hypothesis was accepted; i.e. the two samples were considered not to be different. Upon observation of a *p* value that was less than the chosen significance threshold of 0.05, the null hypothesis was rejected, and the alternate hypothesis was accepted; i.e. the two samples were considered significantly different.

## Results

### Patient demographics

In the present study, 34 patients with 60 Straumann (Straumann Holding AG, Basel, Switzerland) implants were enrolled. The test group included 17 patients with 30 short implants, whilst the control group included 17 patients with 30 standard implants. The mean age of the patients was 56.94 ± 14.74 years in the test group and 60.31 ± 10.46 years in the control group. Table 1 shows the study implant characteristics in the test and control groups. All of the selected patients were classified as “Group I” according to the American Society of Anesthesiology (ASA) [18]. Table 2 shows the ASA classification.

**Table 1:** The relevant properties of the short and standard implants

	**Short**	**Standard**
**Position**		
Front	2	4
Premolar	17	9
Molar	11	17
**Diameter**		
3.3 mm	10	4
4.1 mm	18	26
4.8 mm	2	0
**Type**		
Bone-level	17	16
Tissue-level	13	14
**Material**		
Grade4Ti	26	30
TiZr	4	0
**Surface**		
SLA	23	30
SLActive	7	0

**Table 2:** The ASA classification

ASA PS classification	Definition
Group I	A normal healthy patient
Group II	A patient with mild systemic disease
Group III	A patient with severe systemic disease
Group IV	A patient with severe systemic disease that is a constant threat to life
Group V	A moribund patient who is not expected to survive without the operation
Group VI	A declared brain-deal patient whose organs are being removed for donor purposes

### Radiographic outcomes

**Fig. 1:**
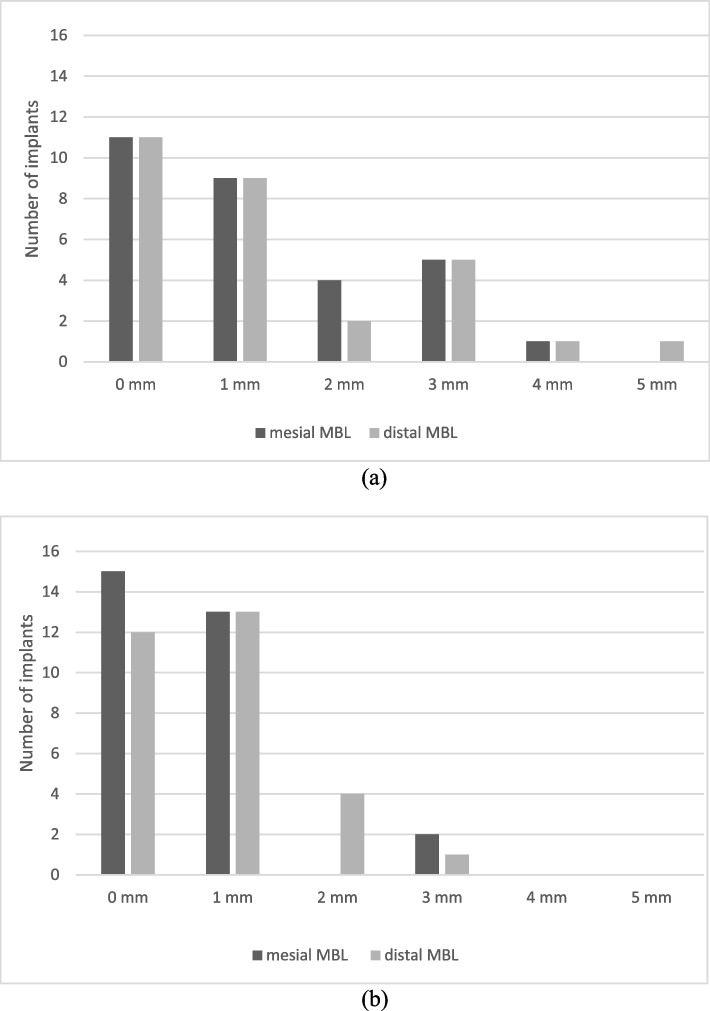
**a** Mean MBL mesially and distally for short implants. **b** Mean MBL mesially and distally for standard implants

The mean MBL was 1.2 ± 1.21 mm mesially and 1.36 ± 1.47 mm distally for short implants (Fig. 1a) compared to 0.63 ± 0.80 mm mesially and 0.78 ± 0.70 mm distally for the standard-length implants (Fig. 1b). The MBL values in the two groups were compared using Welch’s t test at a significance level of 0.05. The two MBL values were compared individually on the mesial and distal sides and using their means. The detailed *p* values of Welch’s t tests are shown in Table 3.

**Table 3:** The *p* values of the statistical tests for MBL

	***p* value**
Mesial	0.038
Distal	0.071
Mean	0.034

In the case of short BL implants, the mean MBL was 1.44 ± 1.41 mm; in the case of short TL implants, the mean MBL was 1.07 ± 1.16 mm. In the case of standard BL implants, the mean MBL was 0.87 ± 0.90 mm; in the case of short TL implants, the mean MBL was 0.46 ± 0.50 mm.

### Clinical outcomes

**Fig. 2:**
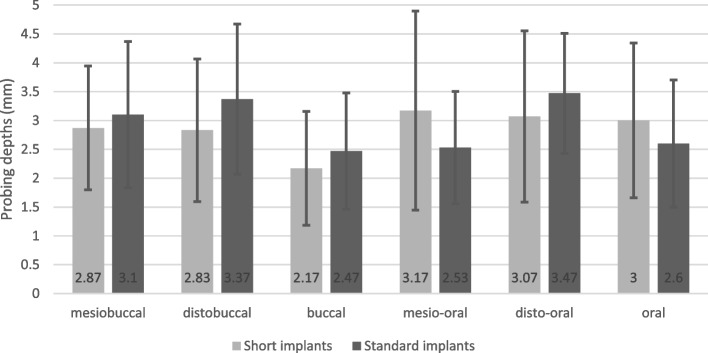
Probing depths around short and standard implants

During the study period, the survival rate of the implants was 100% in the test and control groups. The probing depth values measured in the test group were as follows: mesiobuccally, 2.86 ± 1.07 mm; distobuccally, 2.83 ± 1.23 mm; buccally, 2.16 ± 0.98 mm; mesio-orally, 3.16 ± 1.72 mm; disto-orally, 3.06 ± 1.48 mm; and orally, 3.00 ± 1.33 mm. The probing depth values measured in the control group were as follows: mesiobuccally, 3.10 ± 1.26 mm; distobuccally, 3.36 ± 1.29 mm; buccally, 2.46 ± 1.00 mm; mesio-orally, 2.53 ± 0.93 mm; disto-orally, 3.46 ± 1.04 mm; and orally, 2.6 ± 1.10 mm. Table 4 and Fig. 2 summarise the probing depth values measured around the implants at six characteristic points. The probing depth values in the two groups were evaluated using Welch’s t test at a significance level of 0.05. The six probing depth values were compared (i) in an individually pairwise and (ii) in a mean pairwise fashion. The detailed *p* values of Welch’s t tests are shown in Table 5. None of the above-listed *p* values were below the significance threshold. However, there were significant differences between the probing depth of each tooth group (front, premolar, molar). For the probing depth values, Welch’s t tests resulted in *p* values of 0.027 and 0.016 between the front and molar implants and between the front and premolar implants, respectively. The correlation between the probing depth and the time since prosthetic loading was calculated to be − 0.0759. The correlation between MBL and the time of prosthetic loading was calculated to be 0.0562. Therefore, we could not correlate between the probing depth and MBL, nor the time since loading. In the case of short BL implants, the mean PD was 2.7 ± 0.75 mm; in the case of short TL implants, the mean PD was 2.9 ± 1.18 mm*.* In the case of standard BL implants, the mean MBL was 2.93 ± 0.80 mm; in the case of short TL implants, the mean MBL was 2.85 ± 1.07 mm*.* The *Silness–Löe plaque index* (SLPI) was 0 in 70% of the patients and 1 in 30% of the patients in the test group. The SLPI was 0 in 90% of the patients and 1 in 10% of the patients in the control group. According to the SLPI values, the general oral hygiene of the patients was good. Bleeding on probing was positive in 50% of the patients in the test group and in 30% of the patients in the control group. Table 6 shows the summarised SLPI and BOP values for the test and control groups. The average time period of prosthetic loading was 39.33 ± 21.96 months in the test group and 41.6 ± 27.5 months in the control group. The minimum time since prosthetic loading was 9 months in the test group and 20 months in the control group. The maximum time since prosthetic loading was 95 months in the test group and 134 months in the control group. In the test group, the loading time was more than 2 years in 67% of the cases and longer than 5 years in 30% of the cases. In the control group, the loading time was greater than 2 years in 83.33% of the cases and exceeded 5 years in 13.3% of the cases. Table 7 shows the distribution in percentage of the cases according to the prosthetic loading time. In both the test and the control groups, the patients had fixed prosthetic appliances on the placed implants (single crowns and bridges). None of the patient groups required an axis correction abutment during the fabrication of fixed prosthetic appliances. A prosthetic abutment fracture was not registered.

**Table 4:** Probing depths around short and standard implants

	**Short implant (mm)**	**Standard implant (mm)**
Mesiobuccal	2.86	3.10
Distobuccal	2.83	3.36
Buccal	2.16	2.46
Mesio-oral	3.16	2.53
Disto-oral	3.06	3.46
Oral	3.00	2.60
Average	2.84 ± 0.09	2.91 ± 0.35

**Table 5:** The *p* values of the statistical tests for probing depth

	***p* value**
Mesiobuccal	0.445
Distobuccal	0.108
Buccal	0.248
Mesio-oral	0.076
Disto-oral	0.232
Oral	0.211
Mean	0.762

**Table 6:** The SLPI and BOP values in the test and control groups

	**Test group**	**Control group**
SLPI 0	70%	90%
SLPI 1	30%	10%
BOP	50%	30%

**Table 7:** Distribution in percentage of the cases according to the prosthetic loading time

Prosthetic loading time	Test group	Control group
> 2 years	67%	83.33%
> 5 years	30%	13.3%
0.5–1 year	6%	0%

## Discussion

The MBL values of the two groups were compared (i) individually on the mesial and distal sides and (ii) using their means. Even though the mean MBL demonstrated a true difference between the means of the two samples, the inconsistency in the detailed MBL values did not support this observation. On the one hand, the MBL values on the mesial side demonstrated a significant difference between the groups with short implants and standard implants; on the other hand, the MBL values on the distal sides were comparable. Additionally, the results above were very close to the *p* value threshold of 0.05. These inconsistent MBL results can be explained on the basis of a problem originating from the measurement method. Most notably, deviations in the implant angulation in the oral or the vestibular direction could result in distorted radiographic images; thus, marginal bone loss in particular can appear larger than it is in reality. Together with the observations of the probing depth and the plausible explanation of the measurement errors, these findings indicate that there was no significant difference between the mean MBLs around the short and standard implants.

In our study, there was not a significant difference between the BL and TL implants in the test and control group in terms of mean MBL or PD. This result aligns with the data available in the literature, indicating no difference in terms of MBL between the two implant types [19]; if there is adequate keratinized gingiva around the implant, the success rate is almost the same [20]. Based on in vitro tests, higher stress values can be measured around short BL implants but are not high enough to cause failure [21].

In addition, a significantly greater probing depth on the oral side was recorded, and a correlation with overall oral hygiene habits was demonstrated. This topic is a subject of future studies. Furthermore, the value of the probing depth was expected to be similar to the distance between the marginal bone level and the marginal gingiva [22]; we hypothesised that our study would yield similar results. The probing depth did not differ markedly between the samples. For the probing depth values, Welch’s t tests resulted in *p* values of 0.027 and 0.016 between the front and the molar and between the front and the premolar implants, respectively.

Depending on the position of the implant, a significant difference could be demonstrated between the implants placed in the front region and those placed in the anterior/posterior regions since proper plaque removal in the molar region is more difficult to achieve than in the front region. Moreover, implants may be exposed to different forces in different regions of the jaws, which could also have affected our results. In our study, we could not compare the results of different teeth, but we could evaluate the different tooth groups, thus the front, premolar, and molar teeth.

The correlations between time after prosthetic loading, probing depth, and MBL were evaluated by calculating the respective correlations of the two measured variables with the prosthetic loading time. No correlation was found, indicating that neither the probing depth nor the MBL increased (nor decreased) as a function of time.

The occurrence of bone loss, however, can be explained by three factors during the loading time, as previously shown [23–25]. First, biomechanical forces act on the interfaces between the implant and bone and between the implant and the abutment. These forces are caused by prosthetic loading. Second, bacterial accumulation in microgaps can occur between the implant and the abutment. Consequent inflammatory reactions may be caused by bacterial flora and other inflammatory agents, such as residual cement at the interface. Plaque accumulation and the type of implant–abutment connection can promote an inflammatory response and osteoclast-induced bone resorption. A third factor of peri-implant bone loss may be traumatic surgery. Amongst the most important aspects, subcrestal implant placement, proper implant positioning, a two-phase surgical protocol, and adequate soft-tissue thickness and contouring are recommended. Although soft tissue management often increases the number of surgical procedures, currently, there is increasing effort to reduce surgical loading in periodontology, e.g. with less invasive flap designs [26, 27]. It has been known since Albrektsson and Brånemark [1] published their groundbreaking study in the 1980s that the implant surface plays an important role in osseointegration. Subsequently, different surface treatments have been given greater importance in the long-term success of implants. The interaction of proteins at the nanometric level is emerging as a crucial factor for the integration of implants [28, 29]. Nevertheless, recent studies have shown that with various surface treatments, different osseointegration periods can be achieved [28, 30]. The vast majority of the implants included in our study had an SLA surface. There were seven implants in the test group and zero in the control group with a SLActive surface. A comparison of the two surfaces was thus not possible. The SLActive surface has a more pronounced effect on human bone mesenchymal stem cells [31] and the osseointegration period [32]; however, it has no effect on MBL and the success of the implant in the long term [33].

Osseointegration can also be affected by the material of the implant. Grade 4 Ti and TiZr material implants were found in both examined groups. However, only four implants were made of TiZr in the test group, and zero implants were made of TiZr in the control group. Due to disproportionality, we could not examine this influencing factor. However, implants made of TiZr have a higher modulus of elasticity and greater hardness and thus are more suitable for higher loading [34]; thus, they can have an important role in the use of short implants. The TiZr material also shows better results in terms of MBL than implants made of Ti material [35].

As a result of prosthetic loading, due to biomechanical effects, tension may develop in the bone tissue around the implant. The stress transmission in the bone depends on many factors, such as the length and diameter of the implant. According to in vitro studies, the diameter of the implant is more important than the length of the implant for better stress transmission; however, the length of the implant itself has an effect on the stress generated in the bone [36, 37]. The implant diameter as well as implant co-localization have been identified as further success factors of short implants by Sang-Yun et al. [38] and Tabrizi et al. [39], respectively. In some studies, if wide enough implants were used, then no significant differences were found in the volume of marginal bone resorption or in the implant survival rate according to the length of the implants, surgical type, location of the arch, or prosthetic type [40, 41]. According to the current recommendation of the EDI, if a short implant is used, it should be at least 3.75 mm in diameter [5].

In our study, the primary stability of the implants was not covered. However, the length and diameter of the implant may have an effect on primary stability. According to in vitro studies, in terms of primary stability, the length of the implant has a greater role than the diameter of the implant [42]. A lower primary stability may form with short implants; this statement is especially true in the case of lower bone quality [43].

A limitation of our study is that it did not include the measurement of the implant-to-crown ratio (C/R ratio). However, based on numerous studies, a high C/R ratio alone does not cause clinically significant MBL [44–46].

The imprecise definition of implant success is a general problem in the literature. Many authors use different explanations and different measurement values, rendering comparisons between their research infeasible, firstly because the definition of success itself is ambiguous and secondly because the method for evaluating the success rate is also not defined. The scale of implant quality established by Misch et al. [47] used in our study summarises existing definitions of success and related data. Based on the mesial and distal MBL data, 21 out of the 30 implants were in the *success* group, 8 implants *were in the satisfactory survival* group, and 1 implant was in the *compromised survival* group. Table 8 shows the Implant Quality Scale groups.

**Table 8:** The implant quality scale

Implant Quality Scale group	Clinical conditions
I. Success (optimum health)	No pain or tenderness upon function 0 mobility < 2-mm radiographic bone loss from initial surgery No exudate history
II. Satisfactory survival	No pain on function 0 mobility 2–4-mm radiographic bone loss No exudate history
III. Compromised survival	May have sensitivity on function No mobility Radiographic bone loss > 4 mm (less than1/2 of implant body) Probing depth > 7 mm May have an exudate history
IV. Failure (clinical or absolute failure)	Any of the following: Pain on function Mobility Radiographic bone loss: > 1/2 length of XXX implant Uncontrolled exudate No longer in the mouth

According to the conventions of the scale by Misch, the success rate of the test group in our study was 70%. The success and satisfactory survival rates together yielded 96.66%. Our findings are comparable with other results found in the international literature. In the study of Malmstrom et al. [48], the success rate of short implants was 100% after 2 years of follow-up. Lombardo et al. [49] reported that the success rate of short implants was 97.6% after 3 years of follow-up.

The number of selected patients represents the most significant compromise to the validity of our study. Even though 60 implants were examined in this study, only 34 patients received these implants. This relatively small sample size gives rise to potential success/failure factors in the statistical analysis that remain hidden and therefore cannot be properly measured. However, similar limitations comparable to our research limitations were also identified in the literature. Only systematic reviews feature significantly larger amounts of patient data. Various meta-analyses and systematic reviews indicate that there is no difference between the use of short implants in comparison to standard implants with grafting procedures for the marginal bone level development or success rates [50–52]. However, these findings do not represent the original research results. All the selected patients were classified as “Group I” by the American Society of Anesthesiology classification, but only a small percentage of the overall global population was classified as Group I. However, according to some studies, the general condition does not necessarily significantly affect the long-term success of implants [53].

The use of short implants can have many advantages; however, when using them, it is necessary to take into account what difficulties may arise in the case of a given patient, which may affect the primary stability, the generated tension in the bone, and thus the long-term success of the implant. These factors include bone quality; width of the orovestibular bone (implant diameter); crown-implant ratio; implant location in the jaw; number of implants; harmful behaviours such as bruxism or smoking; and a patient’s general condition and diseases.

## Conclusions

In this study, we found that the long-term success of short implants did not differ widely from the long-term success of standard implants within the limitations of this study’s protocol. These results imply that the advantages of short dental implants can be used with comparable success rates. Our hypothesis was tested in healthy, nonsmoking patients; extrapolation of these results to a more general population is a subject for further research.

## Data Availability

The datasets used and/or analysed during the current study are available from the corresponding author (Kinga Bérczy, berczy.kinga@semmelweis.hu) on reasonable request.

## Abbreviations

EDI: European Association of Dental Implantologists
MBL: Marginal bone loss
BL: Bone level
TL: Tissue level
SLPI: Silness–Löe Plaque Index
BOP: Bleeding on probing
C/R ratio: Implant-to-crown ratio
ASA: American Society of Anesthesiology
